# Effect of off-pump versus on-pump coronary artery bypass grafting in patients with chronic kidney disease

**DOI:** 10.1186/cc10954

**Published:** 2012-03-20

**Authors:** ME Schroeder, L Chawla, Y Zhao, F Lough, F Najam, M Seneff, JM Brennan

**Affiliations:** 1 George Washington University Hospital, Washington, DC, USA; 2 Duke Clinical Research Institute, Raleigh, NC, USA; 3 Duke University Medical Center, Raleigh, NC, USA

## Introduction

Patients with chronic kidney disease (CKD) have been largely excluded from clinical trials of off-pump coronary artery bypass grafting (OPCAB). We sought to determine if the pump status affected outcomes in patients with CKD.

## Methods

Using a nonrandomized cohort of 742,909 nonemergent, isolated CABG cases (including 158,561 OPCAB cases) in the Society of Thoracic Surgery Database from 2004 through 2009, we evaluated the association between pump status (off-pump vs. on-pump) and in-hospital death or incidence of renal replacement therapy (RRT) across strata of preoperative renal function. We used both propensity methods and an instrumental variable (IV) approach to account for imbalances in baseline patient risk.

## Results

Compared with on-pump cases, off-pump cases were of similar age (65.6 vs. 64.9 years) with a similar distribution of preoperative estimated glomerular filtration rate (eGFR). In a propensity weighted analysis, OPCAB was associated with a reduction in composite in-hospital death or RRT, with a progressively increased benefit among those with lower preoperative renal function (eGFR ≥90 ml/minute: risk difference = 0.05 per 100 patients (on-pump minus off-pump), 95% confidence interval = -0.06 to 0.16; 60 to 89 ml/minute: 0.14, 0.05 to 0.23; 30 to 59 ml/minute: 0.66, 0.45 to 0.87; and 15 to 29 ml/minute: 3.66, 2.14 to 5.18). A similar trend was observed for both component endpoints. However, while the IV analysis confirmed the protective effect of OPCAB on composite in-hospital death or RRT among patients with a reduced eGFR, this result was driven by an effect on RRT and not mortality.

## Conclusion

Patients with CKD experience less death or incidence of RRT when treated with off-pump versus on-pump CABG; however, this composite effect is driven by a reduction in incidence of RRT (not death) among low eGFR patients. Prospective trials comparing these procedures in patients with impaired preoperative renal function are warranted.

